# *In silico* biosynthesis of virenose, a methylated deoxy-sugar unique to Coxiella burnetii lipopolysaccharide

**DOI:** 10.1186/1477-5956-10-67

**Published:** 2012-11-15

**Authors:** Gabriela Flores-Ramirez, Stefan Janecek, Ján A Miernyk, Ludovit Skultety

**Affiliations:** 1 Department of Rickettsiology, Institute of Virology, Slovak Academy of Sciences, Dubravska cesta, 9, Bratislava, 845 05, Slovakia; 2 Laboratory of Protein Evolution, Institute of Molecular Biology, Slovak Academy of Sciences, Bratislava, 845 51, Slovakia; 3 USDA, Agricultural Research Service, Plant Genetics Research Unit, Columbia, MO, 65211, USA; 4 Interdisciplinary Plant Group, University of Missouri, Columbia, MO, 65211, USA; 5 Department of Biochemistry, University of Missouri, Columbia, MO, 65211, USA; 6 Centre for Molecular Medicine, Slovak Academy of Sciences, Bratislava, 831 01, Slovakia

**Keywords:** *Coxiella burnetii*, LPS, Deoxysugars, Virenose, Biosynthetic pathway

## Abstract

**Background:**

*Coxiella burnetii* is Gram-negative bacterium responsible for the zoonosis Q-fever. While it has an obligate intracellular growth habit, it is able to persist for extended periods outside of a host cell and can resist environmental conditions that would be lethal to most prokaryotes. It is these extracellular bacteria that are the infectious stage encountered by eukaryotic hosts. The intracellular form has evolved to grow and replicate within acidified parasitophorous vacuoles. The outer coat of *C. burnetii* comprises a complex lipopolysaccharide (LPS) component that includes the unique methylated-6-deoxyhexose, virenose. Although potentially important as a biomarker for *C. burnetii*, the pathway for its biosynthesis remains obscure.

**Results:**

The 6-deoxyhexoses constitute a large family integral to the LPS of many eubacteria. It is believed that precursors of the methylated-deoxyhexoses traverse common early biosynthetic steps as nucleotide-monosaccharides. As a prelude to a full biosynthetic characterization, we present herein the results from bioinformatics-based, proteomics-supported predictions of the pathway for virenose synthesis. Alternative possibilities are considered which include both GDP-mannose and TDP-glucose as precursors.

**Conclusion:**

We propose that biosynthesis of the unique *C. burnetii* biomarker, virenose, involves an early pathway similar to that of other C-3’-methylated deoxysugars which then diverges depending upon the nucleotide-carrier involved. The alternatives yield either the D- or L-enantiomers of virenose. Both pathways require five enzymatic steps, beginning with either glucose-6-phosphate or mannose-6-phosphate. Our *in silico* results comprise a model for virenose biosynthesis that can be directly tested. Definition of this pathway should facilitate the development of therapeutic agents useful for treatment of Q fever, as well as allowing improvements in the methods for diagnosing this highly infectious disease.

## Background

*Coxiella burnetii*, the causative agent of Q fever in humans, is a highly infectious intracellular bacterium that resides in the parasitophorous vacuole of host cells [1]. It causes several outbreaks of this zoonotic disease each year [2,3]. Infected livestock are mainly asymptomatic, but under certain circumstances display infertility, endometritis, placentitis, abortions, stillbirth, and delivery of weak offspring [4-6]. Human Q fever generally results from inhaling infectious aerosols produced by domestic animals, can be either acute or chronic, and exhibits a wide spectrum of clinical manifestations [7-11].

Coxiella have an extracellular matrix similar to that of other Gram-negative bacteria. The outer coat of virulent phase I *C. burnetii* isolates, from natural sources or infections, is critical to evading the host immune system and include full-length lipopolysaccharides (LPS). It includes an O-antigen containing two unique sugars, virenose (6-deoxy-3-C-methyl-D-gulose) and dihydrohydroxystreptose (3-C-(hydroxymethyl) lyxose). These sugars have been used as biomarkers of phase I *C. burnetii* cells and are not present in phase II [12-17]. Serial in vitro passage of *C. burnetii* in either embryonated hen eggs or tissue culture results in cells with morphologically, structurally, and compositionally different from phase I [18,19]. These changes accompany a chromosomal deletion which corresponds to one of the clusters in the genome necessary for O-antigen biosynthesis [20,14]. Two clones, clone I (9Mi/II/C1) and 4 (9Mi/II/C4) of the Nine Mile strains classified as avirulent phase II, have a genomic deletion of 25,997 bp and the third isolate, 9Mi/Baca, which was derived from 9Mi/I by passing for 4,091 days in cell cultures, has a shorter deleted region [20]. The results from sequence analyses indicate that a group of LPS-biosynthetic genes, including genes that encode epimerases, dehydratases, and nucleotide-sugar glycosyltransferases, are part of the deleted segment [14,20].

In our recent comparative proteomics study of phase I and phase II of *C. burnetii*[21], seventeen proteins involved in LPS biosynthesis and metabolism were identified. Nine of these were detected in phase I but not in phase II cells, and are products of genes located in the deleted region of the chromosome. Thus, we confidently proposed these virulence-associated proteins are related to biosynthesis of the LPS I biomarkers. Although, virenose was found to be D-*gulo* enantiomer with the 4C1 ring conformation by NMR spectroscopy [16], the L-form with 1C4 conformation have been reported previously as well [17]. This potential ambiguity prompted us to examine the possible pathways for biosynthesis of the both enantiomers. Herein we propose a homology-based biosynthetic pathway for virenose based upon bioinformatic analyses and supported by the results from prior genomic and proteomic analyses.

## Results and discussion

The 6-deoxysugars constitute a large family of essential components of the LPS of many eubacteria [22]. They are produced by biosynthetic pathways which share early steps [23,24]. During biosynthesis, the monosaccharide-precursors are activated to one of four nucleoside-diphosphate carriers (NDP); adenosine diphosphate (ADP), thymidine diphosphate (TDP), guanosine diphosphate (GDP), or uridine diphosphate (UDP) [22,25] that are then exchanged in the final step by an enzyme which transfers the sugar to a lipid carrier, forming the O-polysaccharide unit. The 6-deoxyhexoses can be synthesized from D-glucose-6-phosphate which is a precursor for the biosynthesis of the TDP, CDP and UDP-sugars, or fructose-6-phosphate which is converted to mannose-6-phosphate and serves as precursor of the GDP-sugars [22,23,25]. The activated precursor NDP-4-keto-6-deoxy-alpha-D-hexose is then transformed by various enzymatic modifications (e.g., epimerization, C- and O-methylation, deoxygenation, amination, ketoreduction, acetylation, dehydrations, etc.) to a great variety of deoxysugars [22,23].

### Glucose-6-phosphate and fructose-6-phosphate as the initial precursors of virenose

The pathway for virenose synthesis depends upon of its enantiomeric and ring conformations. One version involves activation of glucose using TDP, while in the second mannose is activated using GDP. In either instance, the hexose is phosphorylated at C1 before their activation by a nucleotidylyltransferase. 

**Figure 1:**
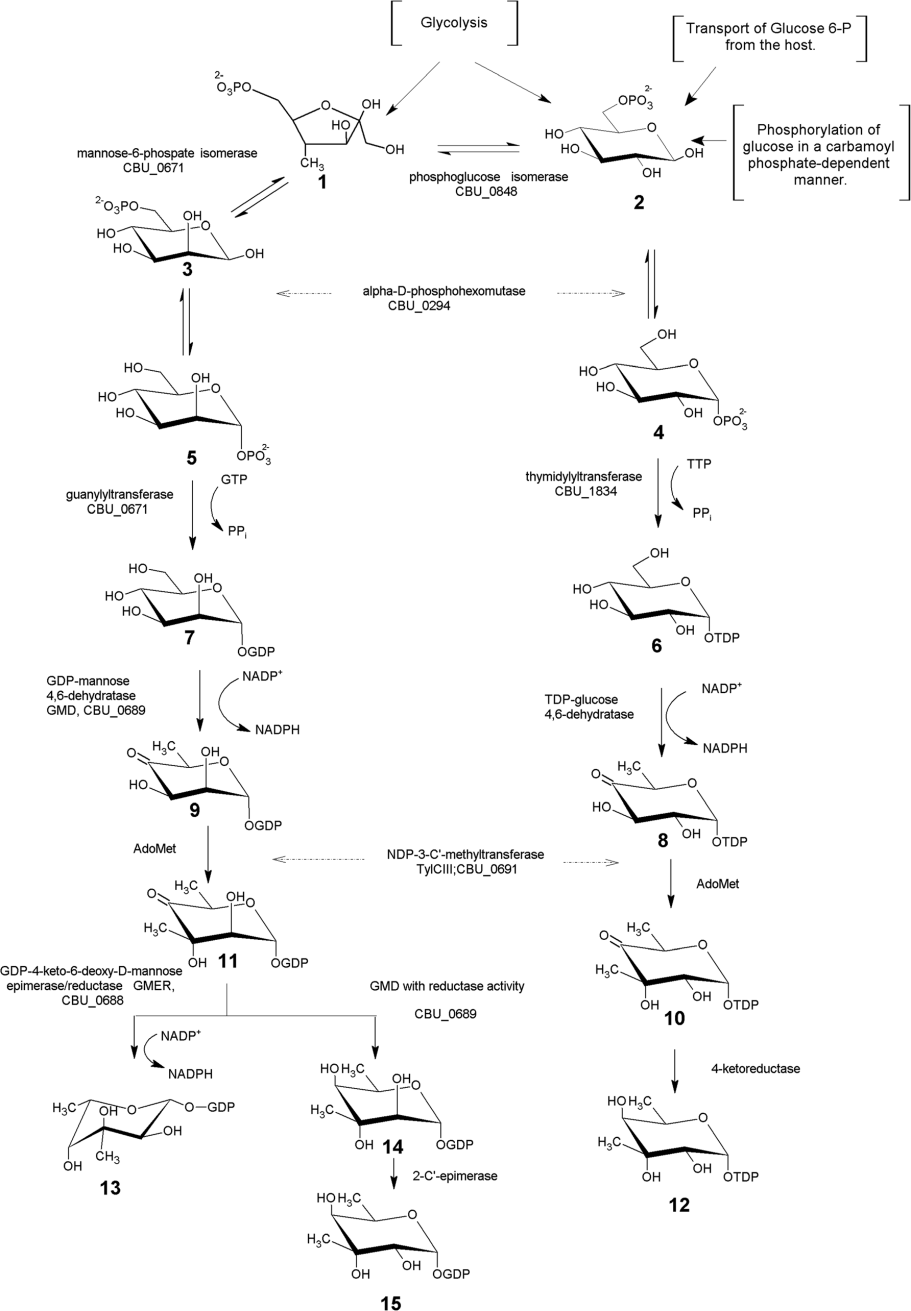
**The proposed pathways of C. burnetii for virenose biosynthesis.** The pathway might begin with either fructose-6-phosphate (**1**) glucose-6-phosphate (**2**) or mannose-6-phosphate (**3**). The hexose-6-phosphates are then converted to either glucose-1-phosphate (**4**) or manose-1-phosphate (**5**) respectively by a dual-specific α-D-phosphohexomutase. Next, thymidylyltransferase or guanylyltransferase generates dTDP-glucose (**6**) or GDP-mannose (**7**), respectively. The activated sugars are transformed to the common intermediates in the biosynthesis of deoxysugars dTDP-4-keto-6-deoxy-D-glucose (**8**) or GDP-4-keto-6-deoxy-D-mannose (**9**). The carbohydrates are then methylated at C3 by the product of the TylCIII gene yielding the corresponding intermediates (**10**, **11**). Finally, the methylated TDP intermediate is reduced by a 4-ketoreductase to form TDP-D-virenose (**12**). In the GDP route the intermediate GDP-3-methyl-4-keto-6-deoxy-D-idose (**11**) is transformed by GDP-4-keto-6-deoxy-D-mannose epimerase/reductase to GDP-L-virenose (**13**) or it can be converted to GDP-D-virenose (**15**) by the activities of a 4-ketoreductase plus a 2-C’-epimerase.

There is biochemical evidence that *C. burnetii* can convert glucose to pyruvate [26-28], however, genome sequence analysis of all six isolates has thus far failed to identify a hexokinase responsible for converting glucose to glucose-6-phosphate (Figure 1) or glucose-6-phosphate and 6-phosphogluconate dehydrogenases [29]. Thus, the first steps of both glycolysis and the pentose phosphate pathway appear missing [29]. This might well explain the low biosynthetic capacity and slow growth rate observed for *C. burnetii*. We speculate that *C. burnetii* phosphorylates glucose via a transphosphorylation reaction involving carbamoyl-phosphate and a phosphatidic acid phosphatase family protein encoded by CBU_1267, as described for the 9Mi/I isolate [29]. There are, of course, other as yet-poorly defined alternatives. Possibly glucose-6-phosphate (2) is obtained from the host cells. Both GDP-mannose, and fructose 6-phosphate (1) or mannose-6-phosphate (3) are potential sources of glucose-6-phosphate, invoking participation of a mannose-6-phosphate isomerase pyrophosphorylase-type or reaction (PMI-GMP; E.C. 5.3.1.8) [30] such as that found as a participant in synthesis of the capsular polysaccharide of *Pseudomonas aeruginosa*, *Salmonella thyphimurium,* and *Xanthomonas campestris*[31,32]. The PMI-GMP enzymes posses separate domains for the mannose isomerase (PMI) and GDP-D-mannose pyrophosphorylase (GMP) activities [33]. A zinc-binding motif and the catalytic amino acid residue R408 are both characteristic of PMI activity [34]. The GMP activity is defined in the N-terminal by the pyrophosphorylase signature sequence, GXGXR(L)-PK [34]. Based on sequence analysis and comparison to *C. burnetii* genome, the PMI-GMP activity might be catalyzed by the product of the CBU_0671 gene, which includes both of these signatures. It shares 45% (E value e-113), 46% (E value 3e-122), and 39% amino acid identity (E value 4e-88) with the PMI-GMP from *Salmonella enterica* LT2 (AAG41744.1), *Escherichia coli* (YP_002413091), and *Helicobacter pylori* (YP_626781) (Additional file 1-A).

### Phosphohexomutase

Both glucose-6-phosphate (2) and mannose-6-phosphate (3) are transformed to the corresponding hexose-1-phosphate by a phosphohexomutase [22,23,25] during biosynthesis of deoxysugars. Phosphomannomutase/phosphoglucomutase (PMM/PGM; E.C. 5.4.2.8) can convert both glucose and mannose, which are C2 epimers. In *C. burnetii*, the product of the CBU_0294 locus has 53% sequence identity (E value e-145) with the PMM/PGM from *P. aeruginosa* (PDB: 1K2Y)[35] (Additional file 1-B). It catalyzes a reversible intramolecular phosphoryl-transfer from C6 to C1 using a Ser108 phospho-enzyme intermediate [35]. The PMM/PGM sequence of *P. aeruginosa* includes the sugar binding motif GEMS(G/A) at positions 324–328 (Additional file 1-B), the catalytic residue is R421, and residues involved in phosphate binding (Y17, K285, R421, S423, N424, and T425) [35]. All of these sequence motifs are conserved in the PMM/PGM sequence of *C. burnetii* (CBU_0294).

### Nucleotide-sugar formation

Both glucose-1-phosphate (4) and mannose-1-phosphate (5) would need to be activated to a NDP-derivate. The activation of D-glucose-1-phosphate (4) to dTDP-D-glucose (6) would employ D-glucose-1-phosphate thymidylyltransferase (G1PTT; EC 2.7.7.24)[36], which has been demonstrated to be involved in the synthesis of L-rhamnose, a common component of the cell wall of many pathogenic bacteria. The *P. aeroginosa* enzyme has been named RmlA (AAG08548) [37], while the *Salmonella typhi* enzyme was designated RfbA (NP_456644) [38]. In the actinobacteria, G1PTT is referred to as TylA1 (AAA2134), and is associated with the synthesis of mycarose, a component of the antibiotic tylosine produced by *Streptomyces fradiae*[36] or eryA when associated with synthesis of erytromycin by *Saccharopolyspora erythraea*[39]. Based on sequence analysis, the corresponding *C. burnetii* gene product (CBU_1834) shares 62% (E value e-105), 61% (E value e-101), and 63% (E value 4e-107) amino acid identity with RfbA, RmlA, and TylA1, respectively. The primary structure of the protein encoded by the G1PTT from *P. aeroginosa* contains the motif (G)GXGXR(L) and the catalytic residues R15, K25, D110, K162 and D225, as well as residues involved in the specificity for thymidine, G10, Q82, and G87, which form hydrogen bonds with the pyrimidine ring. All these residues are conserved in various G1PTT [38] as well as in the product of the CBU_1834 gene (Additional file 1-C), although CBU_1834 is located outside of the O-antigen gene cluster region. It has, however, been previously reported that enzymes integral to specific pathways are often located outside of otherwise common genic-regions [40].

If the pathway includes mannose-1-phosphate (5) as an intermediate, it can be transformed to GDP-α-D-mannose (7) by mannose-1-phosphate guanylyltransferase. Results from BLAST analyses indicate similarity with the product of CBU_0671 and the guanylyltransferase.

### NDP-4-keto-6-deoxy-hexose is the key intermediate

After activation, any of the proposed intermediates will need to be transformed into the key precursor for all NDTP-sugars, an NDP-4-keto-6-deoxy-hexose. This transformation involves dehydratation at C4 and C6 via dNDP-D-hexose 4,6-dehydratase activity [22]. The dTDP-D-glucose (6) might be transformed into dTDP-4-keto-6-deoxy-D-glucose (8) by the TDP-glucose 4,6 dehydratase (TGD). The product of RmlB (EC 4.2.1.46) from *Salmonella enterica* serovar thypimurium [41] was first identified in the L-rhamnose biosynthetic pathway. Homologues of this enzyme have also been found in *Streptomyces venezuelae, E. coli* K12, *P. aeroginosa*, *Pasteurella pseudotuberculosis* and plants [41]. Sequence analysis in *C. burnetii* revealed eight gene products sharing similarity with RmlB (PDB: 1KEU) of *S. typhi*. The extent of sequence identity is similar among all eight proteins, CBU_0677 (27%; E value 2e-23), CBU_0844 (22%; E value 4e-21), CBU_0689 (24%; E value 2e-15), CBU_0829 (25%; E value 1e-14), CBU_0676 (24%; E value 8e-12), CBU_0688 (21%; E value 1e-4), CBU_1837 (22%; E value 2e-4) and CBU_0681 (21%; E value 0.015) (Additional file 1-D). Both CBU_0688 and CBU_0689 were located within the deleted multigenic region thought to be necessary for the synthesis of LPS. At the same time, CBU_0676 and CBU_0677 are close to this region. In contrast, CBU_1837 is near to the thymidylyl transferase gene (CBU_1834). The results from structural studies indicate that the protein is a homodimer that catalyzes a NAD-dependent reaction. The conserved catalytic triad includes YXXXK (residues 167–171 in RmlB) and a conserved motif GXXGXXG at the N-terminus. Both motifs, together with the His residue at position 300 which binds the ribose of dTDP, are conserved in the candidates sequences predicted to have dTDP D-glucose-4,6-dehydratase activity in *C. burnetii.* Moreover RmlB also exhibits both structural and mechanistic similarities to the other NDP-hexose-4,6-dehydratases. In toto, the conservation of characteristics makes it very difficult to make a bioinformatics-based prediction of which specific single gene product is involved in the pathway.

An identical reaction is required to transform the GDP-α-D-mannose (7) into GDP-4-keto-6-deoxy-D-mannose (9) employing the GDP-mannose-4,6-dehydratase (GMD) which activity was biochemically characterized in *Mortierella alpine*[42]. The structure of GMD (PDB: 1RPN) from *P. aeroginosa* was determined recently [43]. This enzyme is involved in the biosynthetic pathway leading to GDP-D-rhamnose [43], a sugar found at trace levels in the LPS of *C. burnetii*[16]. The results from BLAST analyses indicate that CBU_0689 has a 51% (E value e-105) and 50% identity (E value 7e-91) to both GMD from *E. coli* (PDB; 1DB3) [44] and *P. aeroginosa* (PDB; 1RPN)[43], respectively (Additional file 1-E). This enzyme contains a characteristic Gly-rich fingerprint, G9-XR-G-XX-5, along with the catalytic residues T126, E128, Y150, and K154 (Additional file 1-E). In *C. burnetii*, this gene is present in the region deleted from the phase II genome. Furthermore, the diagnostic catalytic T126 is replaced with a Ser residue. However, the T126S change was also found in the GMD sequences from *Aneurinibacillus thermophilus*, *Mycobacterium tuberculosis*, and *Methanobacterium thermoautotrophicum*[43,44].

### Methylation

Because virenose has a C-3’ methyl group, the key precursor NDP-4-keto-6-deoxy-hexose is also likely to be methylated. Among deoxysugars, methylation reaction at this position are mediated by NDP-hexose C-3’methyltransferases (TylCIII; E.C. 2.1.1.-) using S-adenosylmethionine (adoMet) as the methyl donor. A role for this enzyme has been demonstrated in the biosynthesis of L-mycarose, erythromycin, and avilamycin by *S. fradiae*[45],*S. erythraea*[46], and *S. viridochromogenes*[47], respectively. In *C. burnetii*, the product of the CBU_0691 locus has 44% (E value 1e-127), 44% (E value 3e-121) and 42% (E value 7e-119) amino acid identity with TylCIII from *S. fradiae* (AAD41823), EryBIII from *S. erythrae (*YP_001102998), and aviGI from *S. viridochromogenes* (AAK83176), respectively. All these proteins include consensus sequence regions which typify adoMet-dependent methyltransferases [47] (Additional file 1-F). Motif I is a 9-residue sequence with a conserved Gly positioned 5 residues from the N-terminus and an Asp located 17 residues after the C-terminus, and which mediates contact with adoMet [48]. The D-loop, or motif II, contains an acidic residue, either Asp or Glu, whose side-chain hydrogen makes a bond to the ribose-hydroxyl group of AdoMet [49]. Both motifs are present also in the sequence of the product of CBU_0691 of *C. burnetii* (Additional file 1-F). Although this enzyme has only been demonstrated to be active with TDP-sugars, there is no *a priori* reason that it might not be able to accommodate a range of NDP-sugars. The results from mechanistic studies indicate that the methylation reaction catalyzed by TylCIII proceeds with an inversion of hydroxyl groups at C-3’ [45]. Thus, the equatorial methyl group at C-3’ position and the axial C-3’ hydroxyl group that are structural features of D- (12) and L-virenose (13) [16,17] are reversed.

### Reduction and epimerization

In the proposed biosynthetic pathway leading to TDP-D-virenose (12), i.e., the isomer suggested by Toman and Skultety [16] to be present in the LPS of virulent phase I *C. burnetii*, only a reduction at C4 of TDP-3-methyl-4-keto-6-deoxy-D-gulose (10) is necessary. Several 4-ketoreductases are potentially able to provide this reduction. They include the product of the tylCIV locus from *S. fradiae* (AAD41822.1) which is involved in biosynthesis of mycarose[50,51], the product of the mtmU gene from *S. argillaceus* associated with D-oliose and D-olivose biosynthesis [52,53], and StrL which participates in the synthesis of streptomycin [54]. While the sequences of homologues of 4-ketoreductases are broadly conserved, we were unable to find any obvious candidates within the *C. burnetii* proteome. Thus, we speculate that a dual specificity-enzyme is involved*.* Recently it was reported that the enzyme encoded by TylCIII (CAK50784.1) of *S. argillaceus* has 4-ketoreductase activity [53]. The *C. burnetii* protein encoded by the CBU_0691 locus has 30% identity (E value 4e-49) to TylCIII and we propose that this is the relevant 4-ketoreductase.

In contrast, we have identified three different candidates for the 4-ketoreductase that would be involved in the synthesis of GDP-sugars. These include the GDP-4-keto-6-deoxy-D-mannose reductase (RMD) that is involved in several pathways [55-58], the bifunctional GMD that catalyzes both 4,6-dehydratase and reductase reactions leading to GDP-D-rhamnose synthesis in *Klebsiella pneumoniae*, *Anaurinibacillus thermoaerophilus,* and *P. aeruginosa*[55,58], and the bifunctional GDP-4-keto-6-deoxy-D-mannose epimerase/reductase (GMER; E.C. 1.1.1.271), which is responsible for the last two steps of the GDP-L-fucose synthesis in *E. coli*[59] and *M. alpine*[42]. The pathway to GDP-L-virenose (13), i.e., the isomer suggested by Schramek et al. [17] to be present in the LPS of virulent phase I *C. burnetii,* requires both 4-ketoreductase and epimerase activities. The last intermediate, GDP-3-methyl-4-keto-6-deoxy-D-idose (11), must undergo epimerization at positions C-3 and C-5, yielding the L-enantiomer, followed by a 4 keto-reduction catalyzed by a NADPH-dependent GMER [60]. In *C. burnetii*, the product of CBU_0688 shares 45% identity (E value 5e-78) with the well-described dual-activity GMER from *E. coli* (PDB: 1E6U) [60]. The active site of this enzyme includes S107, Y136, and K140 together with the catalytic residues C109 and H179 required for epimerization reaction [60]. These amino acids/positions are conserved in the *C. burnetii* sequence (Additional file 1-G).

Although the GDP-sugar pathway to GDP-L-virenose appears more likely, there remains the possibility that GDP-D-virenose (15) is involved. Starting with the GDP-3-methyl-4-keto-6-deoxy-D-idose (11), a bifunctional GMD could provide both the dehydration and 4-ketoreduction steps leading to GDP-3-methyl-6-deoxy-D-idose (14) [55]. Finally, a 2-C’-epimerase would be required to form GDP-D-virenose (15). It has not yet been possible during the course of our bioinformatics studies to identify a candidate for the latter enzyme within the proteome of *C. burnetii*.

Recently, Narasaki et al. [61] published the results from analysis of the first three steps of a pathway potentially leading to GDP-β-D-virenose biosynthesis; fructose-6-phosphate to mannose-6-phosphate to mannose-1-phosphate to GDP-β-D-mannose. The enzymes involved, phospho-mannose isomerase, phospho-mannose mutase, GDP-mannose pyrophosphorylase, GDP-mannose 4,6-dehydratase, and C-3’methyltransferase, are also part of one branch of our proposed pathway. It is important to consider, however, that while consistent with and fully supporting our proposal these three steps are not unique to biosynthesis of either virenose or dihydrohydroxystreptose [22,25].

## Conclusions

Herein we propose that biosynthesis of the unique *C. burnetii* biomarker, virenose, involves a pathway similar to that of other C-3’-methylated deoxysugars. Two alternative routes are provided that differ primarily in the nucleotide-sugar involved. The alternatives yield either the D- or L-enantiomers of virenose. Both routes require five enzymatic steps, beginning with either glucose-6-phosphate or mannose-6-phosphate. For the pathway starting with glucose-6-phosphate and ending with D-virenose, we propose involvement of α-D-phosphohexomutase, thymidyltransferase, TDP-glucose-4,6-dehydratase, NDP-hexose-3-C-methyltransferase, and an enzyme with 4-keto-reductase activity that cannot be confidently predicted from analysis of the *C. burnetii* genome. Alternatively, starting with mannose-6-phosphate we predict the involvement of α-D-phosphohexomutase, guanyltransferase, GDP-mannose-4,6-dehydratase, NDP-hexose-3-C-methyltransferase, and GDP-4-keto-6-deoxy-D-mannose epimerase/ reductase, leading to L-virenose. Our *in silico* results comprise a model for virenose biosynthesis that is entirely plausible based upon the results of proteomic analyses and which can be directly tested. Definition of this pathway will facilitate the development of therapeutic agents useful for treatment of Q fever as well as allow improvements in the methods for diagnosing this highly infectious disease.

## Methods

### Sequence analyses

The protein sequences were retrieved from the UniProt knowledge database [62] and NCBI [63] using BLAST searches [64] with the PMI-GMP (NCBI:AAG41744.1), PMM/PGM (PDB:1K2Y), G1PTT (NCBI:AAG08548), GMD (PDB:1RPN), TGD (PDB:1KEU), TylCIII (NCBI:AAD41823), and GMER (PDB:1EGU) sequences as queries. Sequences were aligned using Clustal-W2 [65] on the European Bioinformatics Institute’s server (http://www.ebi.ac.uk/). Manual adjustments were made in order to maximize similarities. Structures were retrieved from the Protein Data Bank (PDB)[66].

## Abbreviations

G1PTT: D-glucose-1-phosphate thymidylyl transferase; GMD: GDP-mannose-4,6-dehydratase; GMER: GDP-4-keto-6-deoxy-D-mannose epimerase/reductase; LPS: Lipopolysaccharide; NDP: Nucleostide dieoxy phosphate; PMM/PGM: Phosphomannomutase/phosphoglucomutase; RMD: GDP-4-keto-6-deoxy-D-mannose reductase; TGD: TDP-glucose 4,6 dehydratase; ADP: Adenosine diphosphate; TDP: Thymidine diphosphate; TylCIII: NDP-hexose 3-C-methyltransferase I.

## Competing interests

The authors declare that they have no competing interests.

## Authors’ contributions

LS, SJ were responsible for planning and designing the study; GFR carried out the sequence alignments; GFR, LS, and JAM were responsible for data analysis and manuscript preparation. All authors read and approved the final manuscript.

## Supplementary Material

Additional file 1 **Alignments of amino acid sequences of predicted enzymes implemented in the synthesis of D- and L-virenose.** (**A**) Mannose-6-phosphate isomerase pyrophosphorylase (PMI-GMP). The blue highlighting indicates the pyrophosphorylase signature sequence (N-terminus) while the zinc-binding motif (C-terminus) is green. The R408 of the C-terminal Zn-binding motif is involved in catalysis (in red). (**B**) Dual phosphomannomutase/phosphoglucomutase (PMM/PGM). The residues involved in phosphate binding (Y17 K285 R421 S423 N424 T425) are in blue together with the phosphorylated S108 (according to the structure of P aeruginosa PMM/PGM; (PDB: 1K2Y). The residues involved in sugar binding (GEMS) are green. (**C**) Glucose-1-phosphate thymydylytransferase (G1PTT). The residues participating in catalysis and those responsible for thymidine-specificity are red and yellow respectively. The conserved N-terminal motif GXXXXL is green. (**D**) DTP-D-glucose-4,6-dehydratase (TGD). BLAST analysis against the C burnetii proteome using the TGD from S enterica (PDB:1G1A) gives eight candidates for such activity: CBU_0677 NAD-dependent epimerase/dehydratase family protein; CBU_0844 capsular polysaccharide biosynthesis protein I; CBU_0689 GDP-mannose-46-dehydratase; CBU_0829 NAD-dependent epimerase/dehydratase family protein; CBU_0676 NAD-dependent epimerase/dehydratase; CBU_0688 GDP-fucose synthetase; CBU_1837 putative NAD-dependent epimerase/dehydratase family protein; and CBU_0681 conserved hypothetical protein. The results from structural studies have shown that the latter protein is a homodimer in a NAD-dependent reaction. The conserved catalytic triad includes YXXXK (residues 167–171 in 1KEU) in light blue and a conserved motif GXXGXXG at the N-terminus in green are conserved in C burnetii. The H300 (yellow) which binds the ribose of dTDP is conserved in four of the sequence candidates in C burnetii. (**E**) GDP-mannose-46 dehydratase (GMD). The characteristic Gly-rich fingerprint sequence of GILFNHEGPXRGXXFVTRK which binds NADPH is highlighted in dark green inside this zone R185 is conserved in all GMD sequences previously characterized (yellow). The catalytic residues T126 Glu128 Y150 and K154 are highlighted in light red. The R35 and R43 residues in *P aeroginosa* comprise the RR loop which participates in the tetrameric interface are indicated in blue. (**F**) NDP-hexose 3-C methyltransferase (TylCIII). The motif I characteristic of S-adenosylmethionine (SAM)-dependent methyltransferases is highlighted in light green; the Gly residue of the SAM-binding site is yellow and the motif II sequence is highlighted in blue with emphasis on the conserved Asp residue in yellow. The Asp positioned 17 residues after motif I thought to be important in making a contact with SAM is shown in orange. (**G**) The bifunctional GDP-4-keto-6-deoxy-D-mannose epimerase/reductase (GMER). The catalytic residues S107 Y136 and K140 are light green the residues involved in epimerization C109 and H179 are yellow. The characteristic N-terminal GXXGXXG motif is green. Click here for file
