# Supplementary material for: In silico biosynthesis of virenose, a methylated deoxy-sugar unique to Coxiella burnetii lipopolysaccharide
Source: Proteome Sci. 2012 Nov 15;10:67. doi: 10.1186/1477-5956-10-67 (PMC3539893; doi:10.1186/1477-5956-10-67)
Supplement: Additional file 1 — Alignments of amino acid sequences of predicted enzymes implemented in the synthesis of D- and L-virenose. (A) Mannose-6-phosphate isomerase pyrophosphorylase (PMI-GMP). The blue highlighting indicates the pyrophosphorylase signature sequence (N-terminus) while the zinc-binding motif (C-terminus) is green. The R408 of the C-terminal Zn-binding motif is involved in catalysis (in red). (B) Dual phosphomannomutase/phosphoglucomutase (PMM/PGM). The residues involved in phosphate binding (Y17 K285 R421 S423 N424 T425) are in blue together with the phosphorylated S108 (according to the structure of P aeruginosa PMM/PGM; (PDB: 1K2Y). The residues involved in sugar binding (GEMS) are green. (C) Glucose-1-phosphate thymydylytransferase (G1PTT). The residues participating in catalysis and those responsible for thymidine-specificity are red and yellow respectively. The conserved N-terminal motif GXXXXL is green. (D) DTP-D-glucose-4,6-dehydratase (TGD). BLAST analysis against the C burnetii proteome using the TGD from S enterica (PDB:1G1A) gives eight candidates for such activity: CBU_0677 NAD-dependent epimerase/dehydratase family protein; CBU_0844 capsular polysaccharide biosynthesis protein I; CBU_0689 GDP-mannose-46-dehydratase; CBU_0829 NAD-dependent epimerase/dehydratase family protein; CBU_0676 NAD-dependent epimerase/dehydratase; CBU_0688 GDP-fucose synthetase; CBU_1837 putative NAD-dependent epimerase/dehydratase family protein; and CBU_0681 conserved hypothetical protein. The results from structural studies have shown that the latter protein is a homodimer in a NAD-dependent reaction. The conserved catalytic triad includes YXXXK (residues 167–171 in 1KEU) in light blue and a conserved motif GXXGXXG at the N-terminus in green are conserved in C burnetii. The H300 (yellow) which binds the ribose of dTDP is conserved in four of the sequence candidates in C burnetii. (E) GDP-mannose-46 dehydratase (GMD). The characteristic Gly-rich fingerprint sequence of GILFNHEGPXRGXXFV [file 1477-5956-10-67-S1.doc]

**Add. File 1-A**

*C.burnetii*_CBU_0671 MEECIVPVLLA**GG**V**G**S**RLWP**V**SR**ESYPKQFCKLFDEFSLLQKTAERAKYISKDADLIVVTNYSYYFLCKDQLE-AIGIYNVHYILEPCSRNTAPAIALAA 99

*S.typhimurium*_AAG41744.1 MSNIILPVVMA**GG**A**G**S**RLWP**L**SR**ALYPKQFLSLTSTNSMLQETIIRLEGIEHEYPLFIC-NEEHRFIVAEQLR-QLSIDHSGIILEPDGRNTGPAIALAA 98

*P.putida*_YP_001670794.1 ---MMIPVILS**GG**S**G**S**RLWP**L**SR**KQFPKQFLALTGEHTLFQQTIERLAFEGMDTPIVVC-NKDHKFIVQEQLA-ALKLETQGILMEPFGRNTAPAVAMAA 95

*E.coli*_YP_002413091.1 ---MIIPIIMA**GG**S**G**T**RLWP**L**SR**SLYPKQFLSLINENSLLQETLKRLDGLNCLPPVIVS-NNEHRFIVAEQLR-QFGVDDFQIILEPVGRNTAPAVALAA 95

*H.pylori*_YP_626781.1 --MKIKNILLS**GG**S**G**K**RLWP**L**SR**SLYPKQFLKLFDHKSLFELSFKRNASLVDET-LIVC-NEKHYFLALEEIENEIKNKSVGFLLESLSKNTANAIALSA 96

: ::::** *.****:** :**** * . :::: : * :.: * .: *: ::: : ::*. .:**. *:*::*

*C.burnetii*_CBU_0671 QYACEYIHPDSVLLVLPSDHQLNDHAYFKSTVRAAMNFVERNKLVVFGIEPQSPKTGYGYIEKGD---AVDEGFEVKRFIEKPPLPLAKEFLLQGNFFWN 196

S.typhimurium_AAG41744.1 FHALN-NGYDPVMLVLAADHEIQERKNFIDSIEIAKQFAENDKLVTFGIVPASPETGYGYIKVGE--KIGPDGYKVAAFVEKPHRLLAEEYINSGKYLWN 195

*P.putida*_YP_001670794.1 MKLVN-EGRDELMLVLPADHVIDDQKALQRALALATIAAERGEMVLFGVPATKPETGYGYIRSSQDALLPEGVARVAQFVEKPDEKRAAEFVQAGGYFWN 194

*E.coli*_YP_002413091.1 LKSLE-LHGDHHMLVLAADHAIQDIEAFHAAVLAAEQESVDNKLVTFGIVPTKPETGYGYIKKGE--QVKNSVFKVNSFVEKPDLETAKNYLEQKCYLWN 192

*H.pylori*_YP_626781.1 LMSER----EDLLIVTPSDHLIKDLQAYENAIKKAIDLAQEGFLVTFGVSIEKPNTEFGYIESPN-------GLDVKRFIEKPSLEKAIEFQKSGGFYFN 185

. : ::* .:** :.: :: * . :* **: .*:* :***. : * *:*** * :: : :*

*C.burnetii*_CBU_0671 SGLFLFKAKDYLNKLEKLANDIYVQSTKAFQATEP----NLEFFRVN-KIFDSCRAGSIDYEVMEKTNSAVMLPLRTNWSDLGCWLSVGEVVEGDEAGNV 291

*S.typhimurium*_AAG41744.1 SGMFMFKASVFLNELKKFRPDIYSICETSLLKSES----DLDFIRVNGKVFNCCPSESIDYAVMENTMQAVVVPLDAKWSDVGSWTALWDISKKNENGNI 291

*P.putida*_YP_001670794.1 SGMFLFRASRFLEELKKHDGDIYDTCVLALERSEE----DGDVLSIDEATFACCPDNSIDYAVMEKTQRACVVPMSAGWSDVGCWSSLWEVHEKDDNGNV 290

*E.coli*_YP_002413091.1 SGMFMFKASVYLDELKKFRPDILAACKESLSSAST----DLDFIRLNSDVFAECPDESIDYAVMEKTQDCVVIPLDADWSDIGSWTSLWEISEKDEHENV 288

*H.pylori*_YP_626781.1 SGMFVFQAGVFLDELKKHAPTILKGCERAFESLENTHFFEQKIARLSEKSMQDLEDVSIDIALMQQSHKIKMVGLNAKWSDLGNFNALFEEAANEPKENV 285

**:*:*:* :*::*:* * . :: . : .. :. : *** :*::: :: : : ***:* : :: : : *:

*C.burnetii*_CBU_0671 CYGD--VMVDKCQNCLISSEGRRVVAIGVKDQVIVSTPDALLVIDKAYSQEVKRAVEQMKLQHDIAATEHPRMYRPWGFYEKLAVGADYQVKYLMVNPGS 389

*S.typhimurium*_AAG41744.1 IRGD--VIAKDTDNCYLHSESRLIGTVGVKDLVIVETKDAVLVAHKNNVQDVKNIVEQLKIQNRQEYLHHREVYRPWGSHDAVADGERFRVKHVTVLPGH 389

*P.putida*_YP_001670794.1 TKGD--VVVQDSRNCMIHGNGKLVSVIGLENIVVVETKDAMMIAHKDKVQGVKQMVKTLDEQGRTETQNHLEVYRPWGSYDSVDMGGRFQVKHITVKPGA 388

*E.coli*_YP_002413091.1 SHGD--VINYNSRNNYIYSEGSLISTVGVNNLIIVQTKDALLVAQQDNVQDIKKIVEILKKQKRSEHISHREVYRPWGRYDSVERGDRYQVKRITVKPGE 386

*H.pylori*_YP_626781.1 SLNQTPIFAKESQNNLVFS-HKVSALLGVEDLAIIDTKDALLIAHKDKAKDLKALVSEVEINNQELLQTHTKVYRPWGSYEVLHESGCYKVKILEVKPNA 384

.: :. . * : . :*::: ::.* **::: .: : :* *. :. : * .:***** :: : . ::** : * *.

*C.burnetii*_CBU_0671 S**LSLQLH**RY**R**SEHWIVVSGEAEVVKGESVFRLHSNQSTFIEKGMKHRLSNPGDEPLLIIEVQSGKYLGEDDIVRFDDAYGRVVESTSL--------- 477

*S.typhimurium*_AAG41744.1 AISKQVH*Y*H**R**AEHWVIVSGTARVYIGDENFLLGENESTFIPVGTVHSIENPGKIPLEIIEVQSGTYLHEDDVERS---------------------- 464

*P.putida*_YP_001670794.1 S**LSLQMHHHR**AEHWIVVSGTAEVTCDENVFLLTENQSTYIPIASVHRLRNPGKIPLEIIEVQSGSYLGEDDIERFEDVYGRTSTPVERGVSVKTIAQ 485

*E.coli*_YP_002413091.1 C**LSTQMHHHR**AEHWVVVAGTAKVTCGERTFFVTENESTFIPIGTVHTLENPGKIPLEVIEIQSGVYLGDDDIVRLSDKYGRVEDK------------ 471

*H.pylori*_YP_626781.1 R**LSLQKH**F**HR**SEHWVVISGMASVELDHKMFELQANESTYIPKNTLHRLANYGKIPLIIIEVQVGEYVGEDDIVRVDDDFNRQNQNA----------- 470

:* * * :*:***::::* * * .. * : *:**:* * : * *. ** :**:* * *: :**: *

**Add. File 1-B**

*C.burnetii*_CBU_0294 MAAQGKINQWNVPEIVPATLFRA**Y**DIRGPVTSEALTPGLAYAVGLSIGSEAREQGQKAIVVGRDGRLSGPKLTAALIQGLCETGLAVLNVGLVPTPLVYFATNRLETN**S**GVMVTASHNPG 120

*P.aeruginosa*_1K2Y MSTAKAP-------TLPASIFRA**Y**DIRG-VVGDTLTAETAYWIGRAIGSESLARGEPCVAVGRDGRLSGPELVKQLIQGLVDCGCQVSDVGMVPTPVLYYAANVLEGK**S**GVMLTGAHNPP 112

*:: :**::******** *..::**. ** :* :****: :*: .:.**********:*. ***** : * * :**:****::*:*:* ** :****:*.:***

*C.burnetii*_CBU_0294 HHNGFKIVLNGKTLRSEEIATIRTRILERRFVKGHGAVVDVDIIEDYESYITKHIQLDRPLKVVVDCGNGIAGKVAPALYRKLGCEVVELFCEVDGHFPNHHPDPTIPANLTDLIHKVKE 240

*P.aeruginosa*_1K2Y DYNGFKIVVAGETLANEQIQALRERIEKNDLASGVGSVEQVDILPRYFKQIRDDIAMAKPMKVVVDCGNGVAGVIAPQLIEALGCSVIPLYCEVDGNFPNHHPDPGKPENLKDLIAKVKA 232

.:******: *:** .*:* ::* ** :. :..* *:* :***: * . * ..* : :*:*********:** :** * . ***.*: *:*****:******** * **.*** ***

*C.burnetii*_CBU_0294 TQADLGLAFDGDADRLGIVTDKGEIIWPDRQMMLFSMDVLSRLPGSDIVFDV**K**CSRSLAEIIKKYGGNPVMWRTGHSILKAKLFEIGAPLA**GEMSG**HIFFKDEWFGFDDGIYVGARLLRI 360

*P.aeruginosa*_1K2Y ENADLGLAFDGDGDRVGVVTNTGTIIYPDRLLMLFAKDVVSRNPGADIIFDV**K**CTRRLIALISGYGGRPVMWKTGHSLIKKKMKETGALLA**GEMSG**HVFFKERWFGFDDGIYSAARLLEI 352

:**********.**:*:**:.* **:*** :***: **:** **:**:*****:* * :*. ***.****:****::* *: * ** ********:***:.********* .****.*

*C.burnetii*_CBU_0294 ISQTNQRTSEIFAELPDSVNTPELKLPMTEEKKQPFMQALLKKADFGNAKLITIDGLRVEFEDGWGLI**R**P**SNT**SPYLILRFEADTEEKLKRIQEIFRTQLRMIDNALELPF 471

*P.aeruginosa*_1K2Y LSQDQRDSEHVFSAFPSDISTPEINITVTEDSKFAIIEALQRDAQWGEGNITTLDGVRVDYPKGWGLV**R**A**SNT**TPVLVLRFEADTEEELERIKTVFRNQLKAVDSSLPVPF 463

:** :: :..:*: :*..:.***:::.:**:.* .:::** :.*::*:.:: *:**:**:: .****:*.***:* *:*********:*:**: :**.**: :*.:* :**

**Add. File 1-C**

*C.burnetii_*CBU_1834 --------------MKGIILAGGTGS**R**LYPLTAVIN**K**HLLPIYDKPMIYYPLSVFMLAGIRDILIISTPQSVPLMQDLLKDGSQWGINLSYAI**Q**DQPR**G**LADAFNVGRFFIGNDNVSLIL 106

*S.typhi*_NP_456644 -----------MKTRKGIILAGGSGT**R**LYPVTMAVS**K**QLLPIYDKPMIYYPLSTLMLAGIRDILIISTPQDTPRFQQLLGDGSQWGLNLQYKV**Q**PSPD**G**LAQAFIIGEEFIGNNDCALVL 109

*E.coli_*ABI98983  -----------MKTRKGIILAGGSGT**R**LYPVTMAVS**K**QLLPIYDKPMIYYPLSTLMLAGIRDILIISTPQDTPRFQQLLGDGSQWGLNLQYKV**Q**PTPD**G**LAQAFIIGEEFIGGDDCALVL 109

*P.aeruginosa_*AAG08548 -----------MK-RKGIILAGGSGT**R**LHPATLAIS**K**QLLPVYDKPMIYYPLSTLMLAGIREILIISTPQDTPRFQQLLGDGSNWGLDLQYAV**Q**PSPD**G**LAQAFLIGESFIGNDLSALVL 108

*S.fradiae_*AAA2134 ------MNDRPRRAMKGIILAGGSGT**R**LRPLTGTLS**K**QLLPVYDKPMIYYPLSVLMLAGIREIQIISSKDHLDLFRSLLGEGDRLGLSISYAE**Q**REPR**G**IAEAFLIGARHIGGDDAALIL 114

*L.pneumophila_*AAU26849 .MLFVQRKIESRLDIMKGIILAGGFGT**R**LYPLTKSIS**K**HMIPVYDKPMIYYAISTLMLANIREILIISTEEHLPLYQNLLSDGSQWGISFAYLV**Q**NEPR**G**IAEAFILGEKFIGNDSVCLVL 120

******** *:** * * :.*:::*:********.:*.:***.**:* ***: : :.** :*.. *:.: * * * *:*:** :* .**.: .*:*

*C.burnetii_*CBU_1834 G**D**NIFYMSQLVNKLREVVQHKHGATIFGYYVNNPSEYGVVEFNKEGHAISLDE**K**PKCPKSNYAVTGLYFYDNQVVDIVKHIKPSSRGELEITDVNRVYLDRKQLSVVVLGRGAAWL**D**TGT 226

*S.typhi*_NP_456644 G**D**NIFYGHDLPKLMEAAVNKESGATVFAYHVNDPERYGVVEFDQNGTAVSLEE**K**PLQPKSNYAVTGLYFYDNSVVEMAKNLKPSSRGELEITDINRIYMDQGRLSVAMMGRGYAWL**D**TGT 229

*E.coli_*ABI98983 G**D**NIFYGHDLPKLMDVAVNKESGATVFAYHVNDPERYGVVEFDKNGTAISLEE**K**PLQPKSNYAVTGLYFYDNDVVEMAKNLKPSARGELEITDINRIYMEQGRLSVAMMGRGYAWL**D**TGT 229

*P.aeruginosa_*AAG08548 G**D**NLYYGHDFHELLGSASQRQTGASVFAYHVLDPERYGVVEFDQGGKAISLEE**K**PLEPKSNYAVTGLYFYDQQVVDIARDLKPSPRGELEITDVNRAYLERGQLSVEIMGRGYAWL**D**TGT 228

*S.fradiae_*AAA2134 G**D**NVFHGPGFSSVLTGTVARLDGCELFGYPVKDAHRYGVGEIDSGGRLLSLEE**K**PRRPLE-PGRHRLYLYTNDVVEIARTISPSARGELEITDVNKVYLEQGR-AAHGAGAVVAWL**D**MGT 232

*L.pneumophila_*AAU26849 G**D**NIHQGRGFSELLQNAKSKLNGATVFAYYVDKPQAYGVVEFNEKQQVLSIVE**K**PAQPKSNYAVTGLYFYDNQVIEIAKSLKPSGRGELEITDINQFYLYENKLDVQILGRGFVWL**D**MGT 240

***:. : . : . : *. :*.* * .. *** *::. :*: *** * . . **:* :.*:::.: :.** ********:*: *: . : . * .*** **

*C.burnetii_*CBU_1834 HHSLTEAGQFVKIIEERQGLKLACLEEIAYLKGFITADQLQRLAEAMPKSSYSDYLMNCVQKLQYSLYRPVEEMVWQV 304

*S.typhi*_NP_456644 HQSLIEASNFIATIEERQGLKVSCPEEIAFRKNFINAQQVIKLAGPLSKNDYGKYLLKMVKGL--------------- 292

*E.coli*  HQSLIEASNFIATIEERQGLKVSCPEEIAYRKGFIDAEQVKALAEPLKKNAYGQYLLKMIKGY--------------- 292

*P.aeruginosa_*AAG08548 HDSLLEAGQFIATLENRQGLKVACPEEIAYRQKWIDAAQLEKLAAPLAKNGYGQYLKRLLTETVY------------- 293

*S.fradiae_*AAA2134 HDSLLQAGQYVQLLEQRQGERIACIEEIAMRMGFISAEQCYRLGQELRSSSYGSYIIDVAMRGAAADSRAQ------- 303

*L.pneumophila_*AAU26849 PETLLSASNYICTIEQRQGLKIGCPEEIAWRMKFISDDELLCRARELNKSSYGQYLAELLNHKRY------------- 305

.:* .*.::: :*:*** ::.* **** :* : . : .. *..*:

**Add. File 1-D**

1G1A -----------MKILIT**GGAGFIG**SAVVRHIIKN---TQDTVVNIDKLTYAGNLESLSDISES-NRYNFEHADICDSAEITRIFEQYQPDAVMHLAAESH-VDRSITGPAAFIETNIV 102

CBU_0677 -------MMRKPIAIVT**GGAGFIG**SHMVDLLLDCG---FQVRVIDNLKGGHRRNLEHRANNP---DLTFEIKDICELSAPHPLFEN--VDYVFHFAGIGD-IVPSIENPIDYLQTNVM 102

CBU_0844 ------MEIVKMRTLVT**GCAGFIG**FHLTKRLLARGDHILGLDNLNDYYDVNLKEARLAQLKEF-PHFSFHKLDLADRQGMTDLFQKHLFDTVVHLAAQAG-VRYSLTNPYAYVDSNLV 110

CBU_0689 ---------MRKKAFIT**GITGQDG**SYLAELLLRKDYEVHGMIRRSSSFNTERLSDIYEEKHKENARLFLHYGDITDGLVLNKLIHEIKPHEVYNLAAQSH-VRVSFDIPVYTMETIGL 108

CBU_0829 -----MMDIRGKKFVVI**GGAGLIG**SHTVDRLLQEDV--AEVIIYDNFVRGTRENLAQALRDPR-TKIYDIGGDINQTDILNTALKG--VDGVFHFAALWL-LQCY-EYPRSAFQTNIQ 106

CBU_0676 ------MTKRFDRILVT**GGAGYVG**SALVPQLLELG---YRVTVYDTLFFGDDF---LPKENP---YLNIVEGDIRDTERLKQCFKD--ADAVISLACISN-DASFELDETLSTSINLE 100

CBU_0688 -------MQKDAPIFVT**GHRGLAG**SAILRRLKKQG--------YSSLITR-----------------THQELDLTNKEKVFEFFANNCPEYVFLAAARVGGINDSNLHPVDFIRDNLA 86

CBU_1837 --------MIMETVLVT**GAGGYIG**SVLVPKLLNKG---YHVKAVDRFYFGSDK---LS-QHP---HLELINEDVRRLQ--PSLFTN--VDYVIDLAAVSN-DPSGDIFEKATWEINHQ 95

CBU_0681 MLEHLQDAGTPSRVVVL**GANGFVG**RALCQRLKQEA-------------------------------VNVLELTRQQVDLLNPDATEQLLKQLQASDTLVITAAEAPCKNAAMLYRNVR 87

.: * * * : . :

1G1A GTYALLEVARKYWSALGEDKKNNFRFHHISTDEVYGDLPHPDEVENSVTLPLFTETTAYAPSSP**Y**SAS**K**ASSDHLVRAWRRTYGLPTIVTNCSNNYGPYHFP----EKLIPLVILNALEG 218

CBU_0677 GTVRVLECAR---------AANVKKLVYAASSSCYGLADVPTR-E---------DHPIAP-QYP**Y**ALS**K**YLGEEAAFHWFQVYGLPVNSIRIFNAYGTRVRTTGVYGAVFGVFFKQKLAD 202

CBU_0844 GFAHILEGCR---------HQSVKHLVFASSSSVYGANEKYPFSE---------SDNVDHPIAL**Y**AAS**K**KANELMAHSYAHLFQLPCTGLRFFTVYGPWGRP----DMALFKFTRNLLAD 208

CBU_0689 GTLNILEAIKN------ADNAKEIRFYQASSSEMYGDVKSVPQTES---------TPFNP-RSP**Y**ACA**K**VFAHYQTINYRESYGLHASTGILFNHESPRRGETFVTRKITSGIAKILAGL 212

CBU_0829 GTFNVLETCV---------AQGVKRLVFSSSASVYGDALEEPMTE---------AHPFNS-RTF**Y**GAT**K**IAGEAMATAYHHRYGLPFVGLRYMNVYGPRQDYRGAYIAVIMKMLDALDKG 207

CBU_0676 AFEPMVKAAK---------AAGVKRFIYASSSSVYGVSETKDVTE---------EHPLVP-LTL**Y**NKY**K**GMCEPLLFKHQSPE-FVCVTIRPATLCGYAPRQR--LDLSVNILTNHAVNN 198

CBU_0688 IQWNVIEASFR---------YKVKRLLFLGSSCIYSNDAPRPLKEIYFN-----SGKLEPTNRA**Y**STA**K**IAGIEHCWAYNRQYKTQYLCAMPTNLFGPNDNYDLENGHVVASLISKIHQA 192

CBU_1837 ARVQSATLAK---------QQKVKRYILPSSCSIYGFQKG-AVDE---------TAKTNP-LTT**Y**AKANEKAEKEILPLATDD-FTVTVMRQATVYGYSPRMR--FDLAINGMVYGAWED 192

CBU_0681 MMNVVCEVLQK---------QAIQQVIYISSDAVYADSDQPLTET-----------SVTAPTSLHGVMHLAREMMLQSVCSENNISLAILRPSLLYGAEDPHN---GYGPNRFRRLADNH 184

: .: *. : :

1G1A KP-----LPIYGKGDQIRDWLYVE---DHARALHMVVTEGKAGETYNIGGHNEKKNLDVVFTICDLLD-------EIVPKATSYREQITYVADRPG**H**DRRYAIDAGKISR 313

CBU_0677 KP-----FTVVGDGTQRRDFLYVT---DVARAFLKAAETRKVGETWNLGAG----NPQSINRLVEL----------IG-------GEVEYIPKRPGEPDCTWADISKIKR 283

CBU_0844 KP-----IDVYNHGKMSRDFTYIDDIVDGILLTLDHPPEPNSAYSANQPNPAKSNAPYRIYNIGSNNPILLTNFIAILEKTLNKKAIKNFLPLQPGDVPETYADVSQLEK 313

CBU_0689 EK-----KIYLGNLEAKRDWGYAKDYVEAMWLMLQQDTPDDYVIATGETWSVKELLEYSFNLVNLN---------------WRDFVVIDPKYYRPAEVDLLLGEPKKAKE 302

CBU_0829 QP-----MTLYGDGSQAYDFVYVE---DCAAANICAMKADTVDEYYNVGTG-KRTSILELAKEIQK----------ITG----TSDNIQFLPQGTTFVKNRIGCPKKAAE 294

CBU_0676 NK-----ITVFG-GSQLRPNLHVQDMCDLYKLLLVVPDEKIAGETFNVGYE--NKSIMEIAHIVKN-----IVEEEFPEKAPIDIVTAPTDDIRSY**H**INSDKIKRCLGFE 290

CBU_0688 KEQKKPNFVLWGSGKAKREFLYSDDLAEACCHLMNLPDDIVKSVFGQDDQPPIVNIGSGKEISIYELA------------LLIQDIIGYQGDIIWD**H**SKPDGALTKVMDV 294

CBU_1837 KC-----IPLMRDGTQYRPMVHVQDTTDVMVLLLQADASEINGQIINVGCEEQNYQLQPLGQLIAE----------VVGQKLDEKIAIEWYGDP-D**H**RSYYVSFDKIKRI 285

CBU_0681 ES-----IILFGEGEEQRDHVYIDDVAEIITRVIQRCSRGVLNIATGQVISFKQLAEKVVQLSNNE-----------------VAIQPSPRQGSMP**H**NGYRPFDITDCQK 272

. . : : .

1G1A ELGWKPLETFESGIRKTVEWYLANTQWVNNVKSGAYQSWIEQNYEGRQ-------------- 361

CBU_0677 DLGWEPTITFADGVSRMMSEIGVWHDAPLWNKESIANATATWFKYLGKNKKEKADVAETIPS 345

CBU_0844 DFQYRPRTPLQKGVKNFVEWYLQYFS------------------------------------ 339

CBU_0689 KLGWQPNTSFHKLIKIMLEHDFKSYGVMLPSSNEKQTVEVCIEGLK---------------- 348

CBU_0829 QIGFKAEVGLTEGLQRLIEWRRSHIAEVEQRREVAIS------------------------- 331

CBU_0676 AKYSIEDAVRDLCKAFKAGKLPDSMTNNRYFNVRCIKALEVV--------------- 337

CBU_0688 S-LMQYLGWSAREGLVSGIKKTYQYYLSYERQAIAIEGIEKNA------------------- 332

CBU_1837 LNWQPQWDAAKGAVELIEKLKNNQLQKTAETITLNWYQELEKWHRILQPVIKYDGILNIK- 346

CBU_0681 AFPDFSYTSIEDGLQYSQLKMKVFKKEVL--------------------------------- 301

: . .

**Add. File 1-E**

*C.burnetii*_CBU_0689 ------------MRKKAFITGITGQDGSYLAELLLRKDYEVHGMIR**R**SSSFNTE**R**LSDIYEEKHKENARLFLHYGDITDGLVLNKLIHEIKPHEVYNLAAQSHVRVSFDIPVYTMETIGL 108

*P.aeruginosa*_1RPN ------------MTRSALVTGITGQDGAYLAKLLLEKGYRVHGLVA**R**RSSDTRW**R**LRELGIEG-----DIQYEDGDMADACSVQRAVIKAQPQEVYNLAAQSFVGASWNQPVTTGVVDGL 103

*E.Coli*_1DB3 -------------SKVALITGVTGQDGSYLAEFLLEKGYEVHGIKR**R**ASSFNTE**R**VDHIYQDPHTCNPKFHLHYGDLSDTSNLTRILREVQPDEVYNLGAMSHVAVSFESPEYTADVDAM 107

: *::**:*****:***::**.*.*.***: * ** . *: .: : : . **::* : : : : :*.*****.* *.* .*:: * * . .:

*C.burnetii*_CBU_0689 GTLNILEAIKNADNAKEIRFYQAS**S**S**E**MYGDVKSVPQTESTPFNPRSP**Y**ACA**K**VFAHYQTINYRESYGLHASTGILFNHESPR**RG**ETFVTRKITSGIAKILAGLEKKIYLGNLEAKRDWG 228

*P.aeruginosa*_1RPN GVTHLLEAIR--QFSPETRFYQAS**T**S**E**MFGLIQAERQDENTPFYPRSP**Y**GVA**K**LYGHWITVNYRESFGLHASSGILFNHESPL**RG**IEFVTRKVTDAVARIKLGKQQELRLGNVDAKRDWG 221

*E.Coli_*1DB3 GTLRLLEAIRFLGLEKKTRFYQAS**T**S**E**LYGLVQEIPQKETTPFYPRSP**Y**AVA**K**LYAYWITVNYRESYGMYACNGILFNHESPR**RG**ETFVTRKITRAIANIAQGLESCLYLGNMDSLRDWG 227

*. .:****: : ******:**::* :: * *.*** *****. **::.:: *:*****:*::*..********* ** *****:* .:*.* * :. : ***::: ****

*C.burnetii*_CBU_0689 YAKDYVEAMWLMLQQDTPDDYVIATGETWSVKELLEYSFNLVNLNWR---------------------------DFVVIDPKYYRPAEVDLLLGEPKKAKEKLGWQPNTSFHKLIKIMLE 321

*P.aeruginosa*_1RPN FAGDYVEAMWLMLQQDKADDYVVATGVTTTVRDMCQIAFEHVGLDYR---------------------------DFLKIDPAFFRPAEVDVLLGNPAKAQRVLGWKPRTSLDELIRMMVE 314

*E.Coli*_1DB3 HAKDYVKMQWMMLQQEQPEDFVIATGVQYSVRQFVEMAAAQLGIKLRFEGTGVEEKGIVVSVTGHDAPGVKPGDVIIAVDPRYFRPAEVETLLGDPTKAHEKLGWKPEITLREMVSEMVA 347

.* ***: *:****: .:*:*:*** :*::: : : :.:. * :: :** ::*****: ***:* **:. ***:*. :: ::: *:

*C.burnetii*_CBU_0689 HDFKSYGVMLPSSNEKQTVEVCIEGLK 348

*P.aeruginosa*_1RPN ADLRRVSRE------------------ 323

*E.Coli*_1DB3 NDLEAAKKHSLLKSHGYDVAIALES-- 372

*:.

**Add. File 1-F**

S.*erythrae*_YP_001102998 ---------MGLGKCRICGNHDLESVLHLGDQALTGVFPRNRDEIVPSVPLELVKCAPPGCGLVQLRESADFGLMYNEGYGYRSGIRPFMINHLHGKVAK 91

S.*fradiae*_AAD41823 MPAVPREDQMIISACRVCGNRELLPVLDLGEQALTGVFPRTREETVPSIPLELVKCSPQGCGLVQLRHTPDPGLMYGEGYGYRSGIRPFMIGHLRRKVAA 10

S.*viridochromogenes_*AAK8317 --MSTTGHSTVIDRCRICDNTELLPVLDLGPQALTGVFPRTRGEDVPYVPLELVRCSPGGCELVQLRHTADFGLMYGEGYGYRSSLNRSMADHLRGKVAA 98

*C.burnetii*_CBU_0691 -------------------------MLHLGNQALTGVFPTDATTAITQGPLELVWCE--DSGLLQLNHSYDPAEMYGDNYGYRSGLNQTMIDHLTNKVNY 73

:*.** ******** :. ***** * .. *:**..: * . **.:.*****.:. * .** **

S.*erythrae*_YP_001102998 LRGMVPVGPDDL**VVDIGSNDS**TLLRGYLPDAPKLAGF**D**LVGEKFRDLYPPEADLVTGFFSADAFEERYGERRAKVVTSIAMFYDLPEPMRFMRDVHDILA 191

S.*fradiae*_AAD41823 IRELVDLGPDDL**VLDIGSNDA**TLLKAY-PEGPRLVGI**D**PSGDKFRELYPPHAELIAEYFSRDVFTARFGTRRARVITSIAMFYDLPDPLAFMRDVHDVLA 199

S.*viridochromogenes_*AAK8317 ITGLVDLGPGDL**VVDIGSNDG**TLLAAYPADGPRLVGV**D**PAATVFAASYPPGVELIPDFFAYDLLGG----RRAKVVTSIAMFYDLPRPMEFMREVGRLLT 194

*C.burnetii*_CBU_0691 LERMVTLQDNDV**VVDIGSNDA**TTLKAYSHQKIRRIGI**D**PTGKKFKHHYTDDITLVADFFSEEAYHS-VEINPARIVTSIAMFYDLESPISFAKQVESILA 172

: :* : .*:*:******.* * .* : : *.* . * *. *:. :*: : . *:::********* *: * ::* :*:

S.*erythrae*_YP_001102998 DDGLWLMEQSYLPSMLDAGAY**D**VVCHEHLEYYALAQIEWMAQRVGLKVVDAEITDVYGGSLCAVLAKQG-SGHPVDEAGLERIRAREAAAKLDTMAPYEA 290

S.*fradiae*_AAD41823 DDGIWVMEQSYLPAMLEADAY**D**IVCHEHLEYYALQQIEWMAERAGLTVLRAELTDVYGGSLCVTLARAS-SPHPRDEAGPARIRARETEAKINTMAPFEE 298

S.*viridochromogenes_*AAK8317 DDGIWVTEQSYLPAMLHACAY**D**VVCHEHLDYYGLRQIEWMAERTGLKVVDAELTPVYGGSLSLVLARRG-SSRQVNEPALARIRAGETD------LPYAE 287

*C.burnetii*_CBU_0691 DDGVWHLEQSYMPYMLRLNSY**D**TICHEHLEYYSLSTVKKILETANFRLIDVVTNDINGGSFAVTAAKSTNKTIKSNQLIIDWMLEQEDRMGLNTIHPYAA 272

***:* ****:* ** :** :*****:**.* :: : : ..: :: . . : ***:. . *: . :: : * *:

S.*erythrae*_YP_001102998 FARETERQRDQLLEFLAKSRAEGKLTLGYGASTKGNVILQYCGLTEQDLPCIGEVSPEKSGCYTPGTGIPIVSEEEAKSRRPDQLLVLPWIYRDGFVERE 390

S.*fradiae*_AAD41823 FARRVEHQRDALRDFLDRSRAAGRLTLGYGASTKGNVILQYCGIGERDLPCIGEVSPEKAGRFTPGTGIPIVSEEDAKAMRPDQLLVLPWIYREGFVERE 398

S.*viridochromogenes_*AAK8317 FARRTEESRDRLLEFLTASRDKGLHTLGYGASTKGNVILQYCGLDETLLPCIAEVNEDKFGCYTPGTNIPIVSEEEARALEPDQFLVLPWIYRDAMVARE 387

*C.burnetii*_CBU_0691 FKERVHKHRAELTSLIGALNAAGKKVLGYGASTKGNVVLQFCGFTEKDIPAIAEVNPDKFNCVTPGTHIPIISELEARAMNPDYFLLLPWHFKEGVLRRE 372

* ..... * * .:: . * .***********:**:**: * :*.*.**. :* . **** ***:** :*:: .** :*:*** :::..: **

S.*erythrae*_YP_001102998 QEFLAGGGKLIFPLPRLEVV 410

S.*fradiae*_AAD41823 RDFLAGGGRLVFPLPRLDVV 418

S.*viridochromogenes_*AAK8317 RDFLASGGSLVFPLPTLEVV 407

*C.burnetii*_CBU_0691 KHYMAQGGKFILPFPEVRII 392

:.::* ** :::*:* : ::

**Add. File 1-G**

1E6U MAKQR-VFIA**G**HR**G**MV**G**SAIRRQLEQRGDVELVLRTRDELNLLDSRAVHDFFASERIDQVYLAAAKVGGIVANNTYPADFIYQNMMIESNIIHAAHQNDVNKLLFLG**S**S**C**IYPKLAKQPM 119

CBU_0688 MQKDAPIFVT**G**HR**G**LA**G**SAILRRLKKQGYSSLITRTHQELDLTNKEKVFEFFANNCPEYVFLAAARVGGINDSNLHPVDFIRDNLAIQWNVIEASFRYKVKRLLFLG**S**S**C**IYSNDAPRPL 120

* *: :*::****:.**** *:*:::* .*: **::**:* :.. *.:***.: : *:****:**** .* :*.*** :*: *: *:*.*:.: .*::**********.: * :*:

1E6U AESELLQGTLEPTNEP**Y**AIA**K**IAGIKLCESYNRQYGRDYRSVMPTNLYGPHDNFHPSNS**H**VIPALLRRFHEATAQKAPDVVVWGSGTPMREFLHVDDMAAASIHVMELAHEVWLENTQP- 238

CBU_0688 KEIYFNSGKLEPTNRA**Y**STA**K**IAGIEHCWAYNRQYKTQYLCAMPTNLFGPNDNYDLENG**H**VVASLISKIHQAKEQKKPNFVLWGSGKAKREFLYSDDLAEACCHLMNLPDDIVKSVFGQD 240

* : .*.*****..*: ******: * :***** :* ..*****:**:**:. .*.**:.:*: ::*:*. ** *:.*:****.. ****: **:* *. *:*:*..:: .

1E6U -MLSHINVGTGVDCTIRELAQTIAKVVGYKGRVVFDASKPDGTPRKLLDVTRLHQLGWYHEISLEAGLASTYQWFLENQDRFRG-------- 321

CBU_0688 DQPPIVNIGSGKEISIYELALLIQDIIGYQGDIIWDHSKPDGALTKVMDVSLMQYLGWSAREGLVSGIKKTYQYYLSYERQAIAIEGIEKNA 332

. :*:*:* : :* *** * .::**:* :::* *****: *::**: :: *** . .* :*: .***::*.
